# Comparison between Suspected and Confirmed COVID-19 Respiratory Patients: What Is beyond the PCR Test

**DOI:** 10.3390/jcm11112993

**Published:** 2022-05-25

**Authors:** Stefania Principe, Amelia Grosso, Alida Benfante, Federica Albicini, Salvatore Battaglia, Erica Gini, Marta Amata, Ilaria Piccionello, Angelo Guido Corsico, Nicola Scichilone

**Affiliations:** 1 Department of Pulmonology–Palermo (PA) (Italy), AOUP Policlinico Paolo Giaccone, University of Palermo, 90127 Palermo, Italy; stefania.principe@unipa.it (S.P.); alida.benfante@unipa.it (A.B.); salvatore.battaglia@unipa.it (S.B.); marta.amata@gmail.com (M.A.); ilaria.piccionello@virgilio.it (I.P.); 2 Department of Respiratory Medicine–Amsterdam, Amsterdam UMC, University of Amsterdam, 1105 AZ Amsterdam, The Netherlands; 3 Department of Pulmonology, Fondazione IRCCS Policlinico San Matteo, 27100 Pavia, Italy; amelia.grosso@gmail.com (A.G.); federica.albicini@gmail.com (F.A.); erica.gini@gmail.com (E.G.); corsico@unipv.it (A.G.C.)

**Keywords:** COVID-19, PCR test, COVID-19 diagnosis

## Abstract

COVID-19 modified the healthcare system. Nasal-pharyngeal swab (NPS), with real-time reverse transcriptase-polymerase (PCR), is the gold standard for the diagnosis; however, there are difficulties related to the procedure that may postpone it. The study aims to evaluate whether other elements than the PCR-NPS are reliable and confirm the diagnosis of COVID-19. This is a cross-sectional study on data from the Lung Unit of Pavia (confirmed) and at the Emergency Unit of Palermo (suspected). COVID-19 was confirmed by positive NPS, suspected tested negative. We compared clinical, laboratory and radiological variables and performed Logistic regression to estimate which variables increased the risk of COVID-19. The derived ROC-AUCcurve, assessed the accuracy of the model to distinguish between COVID-19 suspected and confirmed. We selected 50 confirmed and 103 suspected cases. High Reactive C-Protein (OR: 1.02; CI95%: 0.11–1.02), suggestive CT-images (OR: 11.43; CI95%: 3.01–43.3), dyspnea (OR: 10.48; CI95%: 2.08–52.7) and respiratory failure (OR: 5.84; CI95%: 1.73–19.75) increased the risk of COVID-19, whereas pleural effusion decreased the risk (OR: 0.15; CI95%: 0.04–0.63). ROC confirmed the discriminative role of these variables between suspected and confirmed COVID-19 (AUC 0.91). Clinical, laboratory and imaging features predict the diagnosis of COVID-19, independently from the NPS result.

## 1. Introduction

In December 2019, a new Coronavirus, named SARS-CoV-2, was isolated in the respiratory tract cells of humans [1]. On 11 March 2020, the WHO declared the first SARS-CoV-2 outbreak in China as an international public health emergency [2], starting the beginning of the COVID-19 pandemic. COVID-19 infection causes mild or moderate symptoms such as cough, fever, asthenia and sometimes headache and gastrointestinal symptoms, such as vomiting and diarrhea [3]. However, a great proportion of individuals also experienced respiratory symptoms such as dyspnea and respiratory failure suggestive of severe pneumonia that led to access to the Intensive Care Unit or death.

The virus has been isolated in biological respiratory fluids, both through oronasal swabs and bronchoalveolar lavage [4]. The analysis of the nasal-pharyngeal swab (NPS), which uses the real-time reverse transcriptase-polymerase chain reaction (RT-PCR), is considered the gold standard for the diagnosis of COVID-19. The sensitivity and specificity of the RT-PCR technique have been discussed in several studies; Dramé et al. [5], showed that the sensitivity of NPS was under 40% and suggested performing NPSs repeatedly over time, in correlation with patients’ symptoms and other diagnostic tests to avoid missing diagnosis. Conversely, Xiang et al. considered the SARS-CoV-2 antibody tests IgM and IgG as a better diagnostic investigation than the swab [6]. However, during the pandemic, NPS appeared to be the simplest and fastest technique, despite the limitations that could be linked to non-optimal management of the sample or the difficulties related to the procedure. As a matter of fact, NPS can give false-positive and false-negative results. This could be the consequence of various factors, such as cross-contamination with other viruses, unsuitable laboratories and inexperienced operators, but also the viral load, which depends on the days of illness that have passed [7]. Moreover, a systematic review by Rodriguez et al. pointed out the need to perform repeated tests in subjects with a strong suspicion of infection, considering that 54% of COVID-19 positive patients showed a false negative result on the first test with the RT-PCR method [8].

During the COVID-19 pandemic, Italy was one of the most affected countries and the positivity rate and the management of screening tests were heterogeneous among regions, especially during the first outbreak [9,10]. The management of a suspected patient i.e., those who, in addition to typical symptoms, reported a close contact with a positive COVID-19 patient or who were living or traveled through areas at a greater risk of infection (defined as a “red zone”), represented the area of uncertainty leading to the lack of a unified policy to face the emergency [9]. As an example, patients with respiratory symptoms and features of COVID-19 disease that accessed the hospital were transferred to the so-called “grey areas”, in which they received prompt assistance while waiting for the NPS results [11]. This system was established to avoid the spread of COVID-19 in non-COVID-19 units.

Even though the vaccination campaign curbed the spread of SARS-CoV-2 infection, the COVID-19 pandemic steadily continues, and it is arduous to make a prevision about its end. Although the severity of the disease and the number of hospital admissions could potentially be reduced by promoting vaccination strategies, the management of the hospital admissions of COVID-19 patients is still unclear and needs to be further implemented in the long term. An early diagnosis, isolation systems and quarantine of suspected patients are fundamental to controlling the spread of the infection [12]. The introduction in the decision-making of the clinical, radiological and laboratory features to increase the risk of COVID-19 could facilitate the diagnosis and reduce the permanence in the “grey areas”. Assuming that the negative result of the RT-PCR NPS cannot completely rule out COVID-19 diagnosis, we aimed to investigate whether clinical, laboratory and imaging characteristics can improve the reliability of the NPS in differentiating suspected from ascertained COVID-19 cases.

## 2. Materials and Methods

### 2.1. Study Population

This is a retrospective, cross-sectional, multicenter study. The population was divided in confirmed and suspected COVID-19 cases. In the first group, the disease was confirmed by the positivity of NPS from the upper respiratory tract by RT-PCR. In the second group, patients with clinical, radiological and laboratory features suggestive of SARS-CoV-2 infection and negative NPS were included. Data were collected from March to May 2020. Suspected COVID-19 cases were selected from medical records of the Emergency Care Unit of the University Hospital of Palermo, Italy, whereas confirmed COVID-19 cases were retrieved from medical records of the Lung Unit of the Fondazione IRCCS Policlinico San Matteo, University of Pavia, Italy. The study obtained the approval of the Ethical Board of both institutions.

### 2.2. Data Collection

Demographic characteristics, respiratory signs and symptoms at admissions, such as fever, dyspnea and cough, clinical laboratory tests with complete blood count and serum biochemical tests (lactic dehydrogenase (LDH), D-dimer and C-reactive protein (CPR)) were extracted from medical electronic records at the time of the admission to the Emergency Care Unit for suspected COVID-19 cases and at the time of the admission to the Lung Unit for COVID-19-confirmed cases. Chest computed tomographic (CT) scans were carried out for all the patients and were considered suggestive of SARS-CoV-2 infection if they reported the following characteristics: evidence of focal unilateral or diffuse bilateral ground-glass opacities with or without co-existed consolidations [13,14]. Respiratory failure was classified according to blood gas abnormalities [15]. In both units, a PCR-NPS test was performed on all the patients at the moment of the admission. Suspected cases were considered negative for SARS-CoV-2 infection after at least two negative PCR-NPS tests within 48–72 h as well as their consequent admission to a non-COVID Unit.

### 2.3. Statistical Analysis

Clinical, laboratory and radiological variables were summarized using means and Standard Deviations (SD). Normality was assessed by visual inspection of histograms and q-q plots. Continuous baseline variables judged to follow a non-normal distribution were summarized using medians and interquartile ranges. A comparison of those characteristics was performed using Chi-squared or *t*-test for categorical and continuous variables, respectively. A logistic regression model was performed after the selection of the variables considered statistically different (with a *p*-value <0.05) between the two groups to evaluate which exploratory variables were considered to increase or reduce the risk of SARS-CoV-2 diagnosis. The Wald test was used to assess the accuracy of the logistic regression classification. Afterward, a prediction model was performed and, according to the predicted classification, the confusion matrix of the dataset was generated to calculate the sensitivity and specificity of the overall predictive values. From the results of the prediction model, we constructed a receiver operating characteristic (ROC) curve and calculated the area under the curve (AUC) to assess the overall accuracy. A *p*-value < 0.05 was considered statistically significant. The analysis was performed using R studio version 1.1.463 (R Studio Inc., Boston, MA, USA) and R version 3.5.1 (The R Foundation for Statistical Computing, Vienna, Austria).

## 3. Results

We included a total of 153 subjects: 50 belonged to the group of confirmed COVID-19 cases and 103 to the suspected COVID-19 cases. A summary of the general characteristics and the comparison between groups is presented in Table 1. Significant differences were found between suspected and confirmed COVID-19 patients in terms of smoking history, physiological parameters and symptoms, such as the presence/absence of fever, dyspnea, respiratory failure, as well as radiological findings (presence/absence of pleural effusion, CT scan suggestive of SARS-CoV-2 infection) and laboratory exams (CPR, Lymphocytes (%), LDH).

A total of four logistic regression models were performed: the first model included all symptoms and physiological features that resulted in significantly different between the two groups (Supplementary Table S1). In this model, the presence of dyspnea (OR 12.59; CI95%: 1.7–93; *p*-value: 0.013) and the evidence of respiratory failure (OR 8.42; CI95%: 1.32–53.62; *p*-value: 0.024) increased the risk of diagnosis of COVID-19. The second model (Supplementary Table S2), which included only radiological findings, showed that the presence of a CT scan indicative of SARS-CoV-2 infection increased the risk of COVID-19 diagnosis (OR 3.73; CI95%: 1.68–8.28; *p*-value: 0.001), while the presence of pleural effusion reduced the risk (OR 0.24; CI95%: 0.09–0.63; *p*-value: 0.002). In the third model (Supplementary Table S3), which collected laboratory findings, we demonstrated that only CPR could be considered as a potential risk factor for COVID-19 diagnosis (OR 1.02; CI95%: 0.11–1.03; *p*-value: <0.001). A fourth logistic regression model was performed bringing the above-mentioned variables together as it is shown in Table 2. 

**Figure 1:**
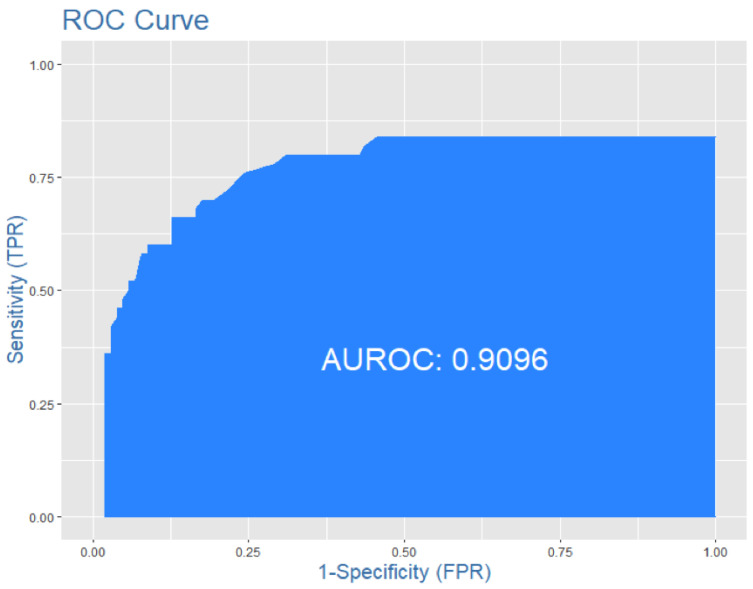
ROC-AUC report the high accuracy of the model in distinguishing the diagnosis of COVID-19 between the two groups.

Those characteristics were able to discriminate between COVID-19-confirmed and COVID-19-suspected cases with a sensitivity of 0.60, specificity of 0.90 and high accuracy, represented with an ROC-AUC curve of 0.91 (Figure 1).

## 4. Discussion

In this study, we were able to prove that a composite measure that includes increased blood levels of Reactive C-Protein, the evidence of suggestive CT-scan alterations and the presence of dyspnea and respiratory failure in addition to the NPS are highly predictive of SARS-CoV-2 infection. The current gold standard to make a diagnosis of active COVID-19 infection should remain the positive detection of viral RNA in respiratory specimens. The use of RT-PCR assay to detect SARS-CoV-2 RNA from the nasopharyngeal tract should remain the preferred initial diagnostic test, although it should be implemented with other evaluations to avoid uncertainties [16]. It has been proven that negative test results do not necessarily rule out the possibility of COVID-19 infection and other complementary measurements, such as bronchoalveolar lavage, should be performed in case of high suspicion of disease [17,18,19,20,21,22]. In such a manner, the diagnostic confirmation of COVID-19 infection can require some days, causing difficulties in terms of management of hospital beds and patients’ follow-up in dedicated units. Suspected SARS-CoV-2 patients generally can stand for days in the “grey areas” of the Emergency Care Unit, receiving assistance while they are waiting for the test results. Therefore, there is the need to implement complementary tools for the decision-making process to accelerate the diagnosis of COVID-19 and facilitate the management of suspected COVID-19 patients. To our knowledge, this is the first study that evaluated, in the real-life scenario of two Italian regions with high and low COVID-19 prevalence, the importance of clinical, radiological and laboratory parameters to implement the diagnosis of COVID-19 and accelerate the decision-making.

As previously demonstrated, CT scan plays a fundamental role in the diagnosis and management of subjects with COVID-19 disease, and it can also be essential in detecting the disease at an early stage [23,24]. In line with these findings, we were also able to demonstrate that imaging could help to detect SARS-CoV-2-infected patients. Specifically, we found that a positive chest CT scan can increase the risk of the presence of SARS-CoV-2 infection, whereas the presence of pleural effusion reduces it. A recent study highlighted the high diagnostic sensitivity of chest CT at certain stages after viral infection [23]. In a study by Xie et al., it was found that all patients presented characteristic CT features of COVID-19 at an early stage, which was confirmed by a positive RT-PCR assay during the isolation period [25]. Similar results were obtained by Ai et al. in a study that evaluated the consistency of chest CT scans in the diagnosis of COVID-19 [26]. CT imaging features of COVID-19 can differ at various disease stages [27,28]. The most frequent CT abnormalities observed in patients with COVID-19 are ground-glass opacities (GGO), usually with multiple and bilateral focal lesions in the posterior and peripheral lung segments, “crazy paving” pattern and less commonly at early stages, pure consolidations [27,29,30,31,32]. In the current study, we were also able to demonstrate that the presence of pleural effusion is indicative of a lower risk of COVID-19 diagnosis. This is in line with the study of Woon et al. [33], who showed that the reported incidence of pleural effusions in COVID-19 pneumonia is low (7.3%). Pleural effusions may occur several days after the onset of symptoms, more likely resembling advanced stages of COVID-19 pneumonia [33]. Therefore, pleural effusion in COVID-19 patients could more likely be considered a more severe radiological evolution of COVID-19 pneumonia [14,34,35,36]. Surely, a chest CT-scan alone is not sufficient to exclude or confirm the diagnosis of COVID-19. Indeed, chest CT-scans should be combined with other relevant clinical information. We proved that patients that reported dyspnea with respiratory failure had an increased risk to be infected by SARS-CoV-2. This is of great importance, given that SARS-CoV-2 infection may result in hypoxemia [37] and the occurrence of respiratory failure increases mortality [38].

In addition to the radiological and clinical findings, we explored other markers able to support the RT-PCR NPS and accelerate the diagnosis of SARS-CoV-2 infection. In this regard, our analysis indicated that increased serum levels of C-reactive protein (CPR) could enhance the risk of SARS-CoV-2 diagnosis. As shown in other studies, the levels of CRP would optimally be rechecked on days 3, 5 and 7 after admission [3,39].

## 5. Conclusions

In conclusion, our findings suggest that NPS tests should be combined with chest CT-scans, clinical symptoms and blood CPR evaluation, to increase the accuracy of the diagnosis of COVID-19 in suspected patients with respiratory symptoms.

## Figures and Tables

**Table 1:** General characteristics and comparison of the two study groups. CT: Computed Tomography; CPR: C-Reactive Protein; LDH: lactic dehydrogenase. Comorbidities are defined as any chronic disease or medical condition.

	COVID-19 Confirmed	COVID-19 Suspected	*p*-Value
N	50	103	
**Age (mean (SD))**	67.02 (10.98)	66.68 (17.52)	0.900
**Sex = M/F n (%)**	33/17 (66.0/34.0)	57/46 (55.3/44.7)	0.279
**Smoking n (%)**			<0.001
Never	18 (47.4)	33 (63.5)	
Former	1 (2.6)	12 (23.1)	
Active	19 (50.0)	7 (13.5)	
**Comorbidities = N/Y n (%)**	12/38 (24.0/76.0)	18/85 (17.5/82.5)	0.462
**Fever = N/Y n (%)**	4/46 (8.0/92.0)	70/33 (68.0/32.0)	<0.001
**Temperature (°C) (mean (SD))**	37.36 (0.82)	36.69 (0.90)	<0.001
**Dyspnea = N/Y n (%)**	7/43 (14.0/86.0)	41/62 (39.8/60.2)	0.002
**Cough = N/Y n (%)**	27/23 (54.0/46.0)	69/34 (67.0/33.0)	0.167
**CT positive = N/Y n (%)**	11/39 (22.0/78.0)	57/46 (55.3/44.7)	<0.001
**Pleural effusion = N/Y n (%)**	44/6 (88.0/12.0)	61/42 (59.2/40.8)	0.001
**D-dimer (ng/mL) (median [IQR])**	558.50 [503.50, 1010.25]	1360.00 [770.00, 3285.50]	0.375
**Lymphocytes (%) (median [IQR])**	10.30 [5.10, 14.20]	13.70 [8.00, 22.55]	0.006
**CPR (mg/L) (median [IQR])**	136.70 [76.15, 203.10]	21.82 [3.56, 67.16]	<0.001
**LDH (mu/mL) (median [IQR])**	436.00 [356.50, 523.75]	257.00 [208.25, 373.50]	<0.001
**Respiratory Failure = N/Y n (%)**	20/30 (40.0/60.0)	70/33 (68.0/32.0)	0.002

**Table 2:** Logistic regression model. OR: Odd ratio; CI(95%): Confidence interval at 95%. CPR: C-Reactive Protein; CT: Chest-Tomography.

	OR	CI(95%)	*p*-Value
**CPR**	1.02	0.11, 1.02	<0.001
**CT positive**	11.43	3.01, 43.3	<0.001
**Pleural effusion**	0.15	0.04, 0.63	0.009
**Dyspnea**	10.48	2.08, 52.7	0.004
**Respiratory Failure**	5.84	1.73, 19.75	0.002

## Data Availability

The data presented in this study are available on request from the corresponding author. The data are no publicly available due to privacy.

## Supplementary Materials

The following supporting information can be downloaded at: https://www.mdpi.com/article/10.3390/jcm11112993/s1, Table S1: Logistic regression model with symptoms and physiological features. OR: Odd ratio; CI(95%): Confidence interval at 95%; Table S2: Logistic regression model with radiological findings. OR: Odd ratio; CI(95%): Confidence interval at 95%. CT: Chest-Tomography; Table S3: Logistic regression model with laboratory findings. OR: Odd ratio; CI(95%): Confidence interval at 95%. CPR: C-Reactive Protein; LDH: Lactic dehydrogenase.

## References

[1] Zhu N., Zhang D., Wang W., Li X., Yang B., Song J., Zhao X., Huang B., Shi W., Lu R. (2020). A Novel Coronavirus from Patients with Pneumonia in China, 2019. N. Engl. J. Med..

[2] WHO/Europe|Coronavirus Disease (COVID-19) Outbreak. https://www.euro.who.int/en/health-topics/health-emergencies/coronavirus-covid-19.

[3] Huang C., Wang Y., Li X., Ren L., Zhao J., Hu Y., Zhang L., Fan G., Xu J., Gu X. (2020). Clinical Features of Patients Infected with 2019 Novel Coronavirus in Wuhan, China. Lancet.

[4] Zhou P., Yang X.L., Wang X.G., Hu B., Zhang L., Zhang W., Si H.R., Zhu Y., Li B., Huang C.L. (2020). A Pneumonia Outbreak Associated with a New Coronavirus of Probable Bat Origin. Nature.

[5] Dramé M., Tabue Teguo M., Proye E., Hequet F., Hentzien M., Kanagaratnam L., Godaert L. (2020). Should RT-PCR Be Considered a Gold Standard in the Diagnosis of COVID-19?. J. Med. Virol..

[6] Xiang F., Wang X., He X., Peng Z., Yang B., Zhang J., Zhou Q., Ye H., Ma Y., Li H. (2020). Antibody Detection and Dynamic Characteristics in Patients with Coronavirus Disease 2019. Clin. Infect. Dis..

[7] Mouliou D.S., Gourgoulianis K.I. (2021). False-Positive and False-Negative COVID-19 Cases: Respiratory Prevention and Management Strategies, Vaccination, and Further Perspectives. Expert Rev. Respir. Med..

[8] Arevalo-Rodriguez I., Buitrago-Garcia D., Simancas-Racines D., Zambrano-Achig P., Del Campo R., Ciapponi A., Sued O., Martinez-García L., Rutjes A.W., Low N. (2020). False-Negative Results of Initial RT-PCR Assays for COVID-19: A Systematic Review. PLoS ONE.

[9] Sartor G., Del Riccio M., Dal Poz I., Bonanni P., Bonaccorsi G. (2020). COVID-19 in Italy: Considerations on Official Data. Int. J. Infect. Dis..

[10] POLITICO–European Politics, Policy, Government News. https://www.politico.eu/.

[11] Scichilone N., Basile L., Battaglia S., Benfante A., Fonte R., Gambino F., Marino S., Messina R., Poma S., Principe S. (2020). Management of Suspected COVID-19 Patients in a Low Prevalence Region. Chron. Respir. Dis..

[12] Hua W., Xiaofeng L., Zhenqiang B., Jun R., Ban W., Liming L. (2020). The Epidemiological Characteristics of an Outbreak of 2019 Novel Coronavirus Diseases (COVID-19) in China. Zhonghua Liu Xing Bing Xue Za Zhi.

[13] Sverzellati N., Ryerson C.J., Milanese G., Renzoni E.A., Volpi A., Spagnolo P., Bonella F., Comelli I., Affanni P., Veronesi L. (2021). Chest Radiography or Computed Tomography for COVID-19 Pneumonia? Comparative Study in a Simulated Triage Setting. Eur. Respir. J..

[14] Shi H., Han X., Jiang N., Cao Y., Alwalid O., Gu J., Fan Y., Zheng C. (2020). Radiological Findings from 81 Patients with COVID-19 Pneumonia in Wuhan, China: A Descriptive Study. Lancet Infect. Dis..

[15] Baudouin S., Turner L., Blumenthal S., Cooper B., Davidson C., Davison A., Elliott M., Kinnear W., Paton R., Sawicka E. (2002). Non-Invasive Ventilation in Acute Respiratory Failure. Thorax.

[16] Patel A., Jernigan D.B. (2020). 2019-nCoV CDC Response Team Initial Public Health Response and Interim Clinical Guidance for the 2019 Novel Coronavirus Outbreak-United States, 31 December 2019–4 February 2020. Am. J. Transplant.

[17] Li Y., Yao L., Li J., Chen L., Song Y., Cai Z., Yang C. (2020). Stability Issues of RT-PCR Testing of SARS-CoV-2 for Hospitalized Patients Clinically Diagnosed with COVID-19. J. Med. Virol..

[18] Kucirka L.M., Lauer S.A., Laeyendecker O., Boon D., Lessler J. (2020). Variation in False-Negative Rate of Reverse Transcriptase Polymerase Chain Reaction–Based SARS-CoV-2 Tests by Time Since Exposure. Ann. Intern. Med..

[19] Winichakoon P., Chaiwarith R., Liwsrisakun C., Salee P., Goonn A., Limsukon A., Kaewpoowat Q. (2020). Negative Nasopharyngeal and Oropharyngeal Swabs Do Not Rule Out COVID-19. J. Clin. Microbiol..

[20] Xiao A.T., Tong Y.X., Zhang S. (2020). False Negative of RT-PCR and Prolonged Nucleic Acid Conversion in COVID-19: Rather than Recurrence. J. Med. Virol..

[21] Young B.E., Ong S.W.X., Kalimuddin S., Low J.G., Tan S.Y., Loh J., Ng O.T., Marimuthu K., Ang L.W., Mak T.M. (2020). Epidemiologic Features and Clinical Course of Patients Infected With SARS-CoV-2 in Singapore. JAMA.

[22] Zou L., Ruan F., Huang M., Liang L., Huang H., Hong Z., Yu J., Kang M., Song Y., Xia J. (2020). SARS-CoV-2 Viral Load in Upper Respiratory Specimens of Infected Patients. N. Engl. J. Med..

[23] Fang Y., Zhang H., Xie J., Lin M., Ying L., Pang P., Ji W. (2020). Sensitivity of Chest CT for COVID-19: Comparison to RT-PCR. Radiology.

[24] Wang S., Kang B., Ma J., Zeng X., Xiao M., Guo J., Cai M., Yang J., Li Y., Meng X. (2021). A Deep Learning Algorithm Using CT Images to Screen for Corona Virus Disease (COVID-19). Eur. Radiol..

[25] Xie X., Zhong Z., Zhao W., Zheng C., Wang F., Liu J. (2020). Chest CT for Typical 2019-NCoV Pneumonia: Relationship to Negative RT-PCR Testing. Radiology.

[26] Ai T., Yang Z., Hou H., Zhan C., Chen C., Lv W., Tao Q., Sun Z., Xia L. (2020). Correlation of Chest CT and RT-PCR Testing in Coronavirus Disease 2019 (COVID-19) in China: A Report of 1014 Cases. Radiology.

[27] Wu J., Wu X., Zeng W., Guo D., Fang Z., Chen L., Huang H., Li C. (2020). Chest CT Findings in Patients with Coronavirus Disease 2019 and Its Relationship With Clinical Features. Investig. Radiol..

[28] Franquet T. (2011). Imaging of Pulmonary Viral Pneumonia. Radiology.

[29] Song F., Shi N., Shan F., Zhang Z., Shen J., Lu H., Ling Y., Jiang Y., Shi Y. (2020). Emerging 2019 Novel Coronavirus (2019-NCoV) Pneumonia. Radiology.

[30] Ng M.Y., Lee E.Y.P., Yang J., Yang F., Li X., Wang H., Lui M.M.S., Lo C.S.Y., Leung B., Khong P.L. (2020). Imaging Profile of the COVID-19 Infection: Radiologic Findings and Literature Review. Radiol. Cardiothorac. Imaging.

[31] Zu Z.Y., Di Jiang M., Xu P.P., Chen W., Ni Q.Q., Lu G.M., Zhang L.J. (2020). Coronavirus Disease 2019 (COVID-19): A Perspective from China. Radiology.

[32] Pan F., Ye T., Sun P., Gui S., Liang B., Li L., Zheng D., Wang J., Hesketh R.L., Yang L. (2020). Time Course of Lung Changes at Chest CT during Recovery from Coronavirus Disease 2019 (COVID-19). Radiology.

[33] Chong W.H., Saha B.K., Conuel E., Chopra A. (2021). The Incidence of Pleural Effusion in COVID-19 Pneumonia: State-of-the-Art Review. Hear. Lung.

[34] Bernheim A., Mei X., Huang M., Yang Y., Fayad Z.A., Zhang N., Diao K., Lin B., Zhu X., Li K. (2020). Chest CT Findings in Coronavirus Disease-19 (COVID-19): Relationship to Duration of Infection. Radiology.

[35] Xiong Y., Sun D., Liu Y., Fan Y., Zhao L., Li X., Zhu W. (2020). Clinical and High-Resolution CT Features of the COVID-19 Infection: Comparison of the Initial and Follow-up Changes. Investig. Radiol..

[36] Guan C.S., Lv Z.B., Yan S., Du Y.N., Chen H., Wei L.G., Xie R.M., Chen B.D. (2020). Imaging Features of Coronavirus Disease 2019 (COVID-19): Evaluation on Thin-Section CT. Acad. Radiol..

[37] Yi Y., Lagniton P.N.P., Ye S., Li E., Xu R.H. (2020). COVID-19: What Has Been Learned and to Be Learned about the Novel Coronavirus Disease. Int. J. Biol. Sci..

[38] Haimovich A.D., Ravindra N.G., Stoytchev S., Young H.P., Wilson F.P., van Dijk D., Schulz W.L., Taylor R.A. (2020). Development and Validation of the Quick COVID-19 Severity Index: A Prognostic Tool for Early Clinical Decompensation. Ann. Emerg. Med..

[39] Chen L.D., Zhang Z.Y., Wei X.J., Cai Y.Q., Yao W.Z., Wang M.H., Huang Q.F., Zhang X. (2020). Bin Association between Cytokine Profiles and Lung Injury in COVID-19 Pneumonia. Respir. Res..

