# Supplementary material for: Comparison between Suspected and Confirmed COVID-19 Respiratory Patients: What Is beyond the PCR Test
Source: J Clin Med. 2022 May 25;11(11):2993. doi: 10.3390/jcm11112993 (PMC9181151; doi:10.3390/jcm11112993)
Supplement: Supplementary file 1 [file jcm-11-02993-s001.zip › jcm-1729707-supplementary.pdf]

Comparison between Suspected and Confirmed COVID-19 Respiratory Patients: What Is beyond the PCR Test

Stefania Principe [1,2], Amelia Grosso [3], Alida Benfante [1], Federica Albicini [3], Salvatore Battaglia [1], Erica Gini [3], Marta Amata [1], Ilaria Piccionello [1], Angelo Guido Corsico [3] and Nicola Scichilone [1,*]

1 *Department of Pulmonology–Palermo (PA) (Italy), AOUP Policlinico Paolo Giaccone, University of Palermo, 90127 Palermo, Italy*

2 *Department of Respiratory Medicine–Amsterdam, Amsterdam UMC, University of Amsterdam, 1105 AZ Amsterdam, The Netherlands*

3 *Department of Pulmonology, Fondazione IRCCS Policlinico San Matteo, 27100 Pavia, Italy*

Supplementary Material

Table S1. Logistic regression model with symptoms and physiological features. OR: Odd ratio; CI(95%): Confidence interval at 95%.

	**OR**	**CI (95%)**	**P-value**
**Smoking History**			
Never	0.87	(0.5, 7. 8)	0.402
Ex	0. 95	(0.05,17.48)	0.974
Active	4.14	(0.45,38.47)	0.211
**Fever**	12.4	(0.12,17.5)	0.993
**Dyspnea**	12.59	(1.7,93)	0.013
**Respiratory Failure**	8.42	(1.32,53.62)	0.024

Table S2. Logistic regression model with radiological findings. OR: Odd ratio; CI(95%): Confidence interval at 95%. CT: Chest-Tomography

	**OR**	**CI (95%)**	**P-value**
**CT- Positive**	3.73	(1.68,8.28)	0.001
**Pleural effusion**	0.24	(0.09,0.63)	0.002

Table S3. Logistic regression model with laboratory findings. OR: Odd ratio; CI(95%): Confidence interval at 95%. CPR: C-Reactive Protein; LDH: Lactic dehydrogenase.

	**OR**	**CI (95%)**	**P-value**
**Lymphocytes**	0.99	(0.94,1.05)	0.886
**CPR**	1.02	(0.11,1.02)	< 0.001
**LDH**	1.01	(0.99,1.03)	0.423
